# Evaluation of the relationship between vitamin D level and adropin, IL-1β, IL-6, and oxidative status in women

**DOI:** 10.55730/1300-0144.5424

**Published:** 2022-05-07

**Authors:** Mehmet ZORLU, Abdüsselam ŞEKERCİ, Muhammed TUNÇ, Eray Metin GÜLER, Bedia GÜLEN, Cumali KARATOPRAK, Muharrem KISKAÇ, Mustafa ÇAKIRCA

**Affiliations:** 1 Department of Internal Medicine, Faculty of Medicine, Bezmialem Vakıf University, İstanbul, Turkey; 2 Department of Medical Biochemistry, Hamidiye Medical Faculty, University of Health Sciences Turkey, İstanbul, Turkey; 3 Department of Emergency, Faculty of Medicine, İstanbul Medipol University, İstanbul, Turkey

**Keywords:** Vitamin D, adropin, IL-1 beta, IL-6, oxidative stress

## Abstract

**Background/aim:**

Vitamin D, adropin, proinflammatory cytokines, and oxidative stress closely related with metabolic homeostasis and endothelial dysfunction. The aim of the present study is to investigate how vitamin D levels affect serum adropin, IL-1ß, IL-6, and oxidative stress.

**Materials and methods:**

A total of 77 female subjects were divided into 3 groups according to vitamin D levels. Biochemical parameters, adropin, IL-1ß, IL-6, oxidative stress markers were studied in these groups, and the results were compared statistically.

**Results:**

Serum adropin, IL-1ß, IL-6, total oxidant status (TOS) and total antioxidant status (TAS) and oxidative stress index (OSI) levels differed significantly between the vitamin D groups (p < 0.05). A significant positive correlation was detected between vitamin D, and adropin and TAS (r = 0.807; p < 0.001, r = 0.814; p < 0.001, respectively). A significant negative correlation was detected between vitamin D, and IL-1ß, IL-6, TOS, OSI (r = −0.725; p < 0.001, r = −0.720; p < 0.001, r = −0.238; p = 0.037, r = −0.705; p < 0.001, respectively).

**Conclusions:**

Vitamin D could show its effects through vitamin D receptors on tissues or on the ENHO gene in adropin secreting tissues via direct or indirect mechanisms. Proinflammatory cytokines, oxidative stress, and adropin targeted studies could contribute to the prevention and treatment of diseases associated with vitamin D deficiency in future.

## 1. Introduction

While vitamin D is mainly synthesized in the skin under the influence of sunlight in humans, a small portion is obtained from foods. Vitamin D mainly regulates calcium, phosphorus and bone metabolism, and is also known as an immunomodulator hormone [1–3]. Vitamin D deficiency might be due to factors including poor sunlight exposure, insufficient intake of vitamin-containing foods and malabsorption syndromes (Crohn’s disease and Celiac disease). Vitamin D has been shown to exert its physiological effects via vitamin D receptors (VDR) present in many tissues and organs [1,4]. Being a worldwide health issue, vitamin D deficiency and insufficiency was associated with many illnesses including osteoporosis, metabolic syndrome, cardiovascular diseases, diabetes, hypertension, endothelial dysfunction, infectious diseases, autoimmune disorders, cancers, and neurological disorders [1,2,5].

Adropin is a peptide hormone coded by the energy homeostasis-associated gene (ENHO) mainly in the liver and brain. It is closely related to glucose and lipid homeostasis and insulin resistance. It is produced in many tissues and organs such as the liver, brain, heart, kidney, pancreas, and gastrointestinal system [6–9]. Adropin has been determined to increase NO release by stimulating endothelial nitric oxide synthase (eNOS) via the interactions between intracellular pathways, improving endothelial cell functions, and protecting the cardiovascular system by providing neovascularization [7,10]. Many studies have reported that serum adropin levels might be associated with obesity, diabetes mellitus, hypertension, cardiovascular diseases, endothelial dysfunction, metabolic syndrome, and several cancers [10–13].

Oxidative stress (OS) is a result of impaired balance between the reactive oxygen species (ROS) formation and antioxidant defense mechanisms. Free radical production occurs continuously in all cells as a part of normal cellular functioning. High amounts of free radicals and ROS formed in tissues due to OS interact with intracellular molecules and cause cellular damage by injuring various biological molecules including proteins, lipids, and nucleic acids [14,15]. Excessive endogenous or exogenous free radical production might have a role in many illnesses. Antioxidants prevent free radical-induced tissue damage by preventing the production of or removing the free radicals. While total oxidant status (TOS) reflects the total effect of all oxidants present in plasma and body fluids, total antioxidant status (TAS) reflects the total effect of all antioxidants. Measurement of TOS or TAS is preferred to individual measurements of serum (or plasma) concentrations of different oxidant or antioxidant molecules [16,17]. OS increase leads to the development of metabolic syndrome, endothelial dysfunction, hypertension, diabetes mellitus, cardiovascular diseases, cancer, and kidney and neurological diseases [14,15,18–21].

Interleukin-1 beta (IL-1β) and interleukin-6 (IL-6) are proinflammatory cytokines especially produced by myelomonocytic cells such as monocytes, macrophages, and dendritic cells. They can be the pathogenic precursor to autoinflammatory, autoimmune, infectious, and degenerative diseases by affecting many cells and organs [22,23]. Moreover, they are associated with endothelial dysfunction, metabolic syndrome, insulin resistance, hypertension, diabetes mellitus, oxidative stress, and cardiovascular diseases [18,24–26].

Many chronic illnesses including endothelial dysfunction, diabetes mellitus, cardiovascular diseases, malignancy, autoimmune disorders which, as shown in the studies, were associated with vitamin D were also similarly associated with adropin, IL-1β, IL-6 and oxidative stress. In the literature search we performed, there were limited number of studies individually evaluating the relationship between vitamin D level, and oxidative status or proinflammatory cytokines (IL-1β,IL-6) in some diseases, however, there was no study evaluating the relationship between vitamin D and adropin or evaluating these parameters together. We aimed to evaluate the relationship between the levels of vitamin D and adropin, IL-1β, IL-6 and oxidative status, which, we think might affect the pathological and metabolic conditions seen in vitamin D deficiency.

## 2. Materials and methods

### 2.1. Study group

This cross-sectional, case-controlled trial was initiated after approval of Bezmialem Foundation University, ethics committee. Our study included 77 female subjects aged 18 to 65 years who consulted to Bezmialem Foundation University, Internal Diseases Outpatient Clinic between July 2020 and December 2020, did not have a known history of chronic illness, were not pregnant or lactating, did not have a history of surgical operation within the last 6 months, did not use antioxidant medication, vitamin supplement, lipid-lowering agent, tobacco or alcohol, did not use vitamin D within the last 3 months, did not do heavy exercise recently and have normal C-reactive protein (CRP) levels. All study subjects gave written informed consent. The subjects were divided into three groups by their vitamin D level (G1: Vitamin D < 20 ng/mL, deficiency; G2: Vitamin D = 20–30 ng/mL, insufficiency; G3: Vitamin D > 30 ng/mL, normal) [5].

### 2.2. Blood assay

Venous blood samples were taken from all subjects into gel tubes between 8:00 a.m. and 9:00 a.m. following 12 h of fasting for biochemical parameters, adropin, TAS, TOS, IL-1β and IL-6 tests, and centrifuged at 3600 rpm for 10 min and the sera were separated. The sera of all subjects were transferred into Eppendorf tubes and kept at −80 °C until the study day.

All volunteers underwent a thorough physical examination with their height and weight being recorded. Weight and height were rounded to the nearest kg and cm, respectively, and body mass index (BMI) was calculated [BMI = weight/(height)^2^].

#### 2.2.1. Measurement of total antioxidant status and total oxidant status (TAS and TOS) in serum

Total oxidant status and total antioxidant status were determined by a recently developed method, colorimetric assay [16]. The TAS results are expressed in mmol Trolox Equiv./L., and TOS in μmol H_2_O_2_ Equiv./L. Coefficients of variation values were less than 10%.

#### 2.2.2. Oxidative stress index (OSI) determination

OSI was determined as TOS/TAS ratio (resulting TAS unit was changed to μmol/L) and calculated as follows: OSI (arbitrary unit) = TOS, μmol H_2_O_2_ Equiv./L/TAS, and mmol Trolox Equiv./L [27].

#### 2.2.3. Measurement of Adropin, IL-1β, and IL-6

Concentrations of adropin, IL-1β, and IL-6 in the serum were measured by a specific commercial ELISA kit according to the manufacturer’s instructions (Adropin: E3231Hu, IL-1β: E0143Hu, IL-6: E0090Hu–Bioassay Technology Laboratory, China). Concentrations were determined with a spectrophotometric microtiter plate reader (Varioskan Flash Multimode Reader, Thermo, Waltham, USA) at 450 nm optical density [27].

### 2.3. Statistical analysis

IBM SPSS (Statistical Package for Social Sciences) statistics 22.0 software was used for the statistical analyses for the study. While evaluating the study data, descriptive statistical methods (mean, standard deviation, median, frequency) were used. Skewness and kurtosis values were used together with the Shapiro-Wilk test to evaluate the normal distribution of the data. While the one-way ANOVA test was used to compare more than two normally distributed variables, the Kruskal Wallis test was used to evaluate more than two nonnormally distributed variables. Tukey and Games Howell tests were used for post-hoc pairwise comparison of the parameters that were significant after ANOVA and the Kruskal Wallis test. For the assessment of correlation between the data, Pearson’s correlation analysis was used for the normally distributed data and Spearman’s correlation analysis for the nonnormally distributed data. Results were evaluated within a 95% confidence interval with significance at a p level of <0.05.

## 3. Results

The mean age of the 77 women participating in the study was 33.79 ± 10.15 years and the mean BMI (body mass index) was 24.90 ± 5.20 kg/m2. The mean age of the groups that we divided into three groups according to their vitamin D levels (G1: Vitamin D < 20 ng/mL; G2:Vitamin D 20–30 ng/mL; G3:Vitamin D > 30 ng/mL) was G1:32.35 ± 10.67, G2:35.14 ± 10.85, and G3:35.41 ± 7.84 were determined. The mean BMI of the three groups was G1:24.41 ± 5.26, G2:26.70 ± 5.79, and G3:23.79 ± 3.81. There was no statistically significant difference between the groups in terms of age and BMI (p: 0.459, p: 0.163, respectively) (Table 1).

There was no statistically significant difference between three groups in terms of glucose, creatinine, alanine aminotransferase (ALT), aspartate aminotransferase (AST), total cholesterol (TC), triglyceride (TG), low-density lipoprotein-cholesterol (LDL-C), high-density lipoprotein-cholesterol (HDL-C), alkaline phosphatase (ALP), gamma glutamyl transferase (GGT), calcium, magnesium, phosphorus, thyroid stimulating hormone (TSH) and free tetraiodothyronine (fT4) (p > 0.05). As for adropin, IL-1β, IL-6, TOS, TAS, OSI, and parathyroid hormone (PTH) levels, a statistically significant difference was detected between the groups (p < 0.05) (Table 1).

**Figure 1:**
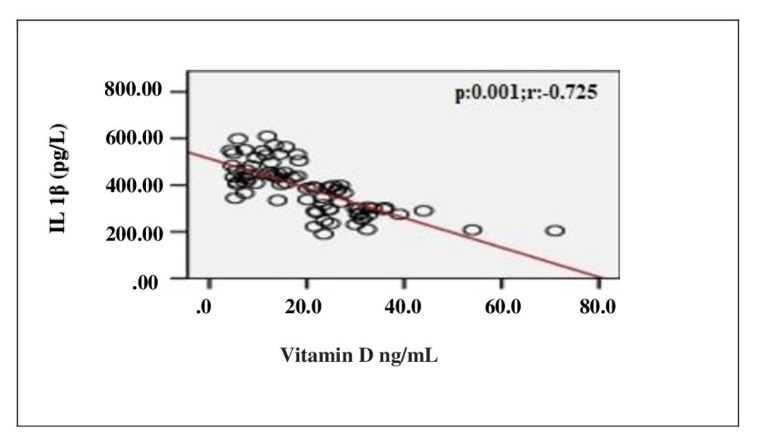
Correlation assessment between vitamin D level, and IL-1β level (n = 77, total subjects). r: Correlation coefficient; IL-1β: Interleukin-1 beta.

**Figure 2:**
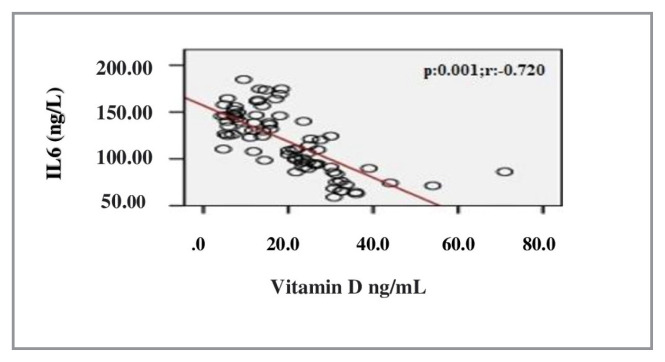
Correlation assessment between vitamin D level and IL-6 level (n = 77, total subjects). r: Correlation coefficient; IL-6: Interleukin-6.

**Figure 3:**
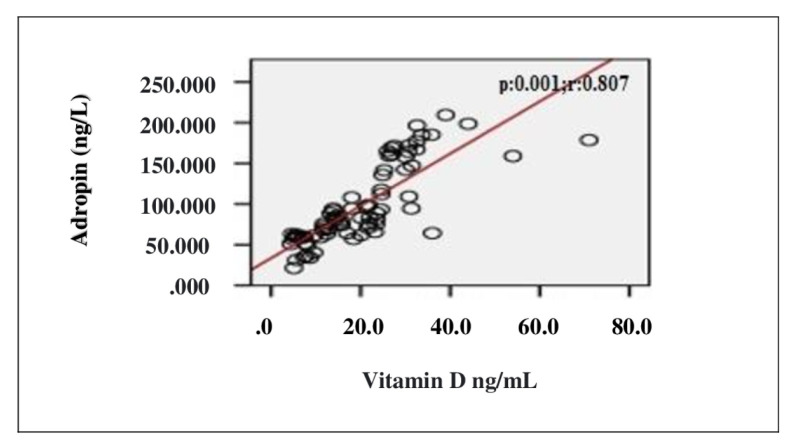
Correlation assessment between vitamin D level and adropin level (n = 77, total subjects). r: Correlation coefficient.

**Figure 4:**
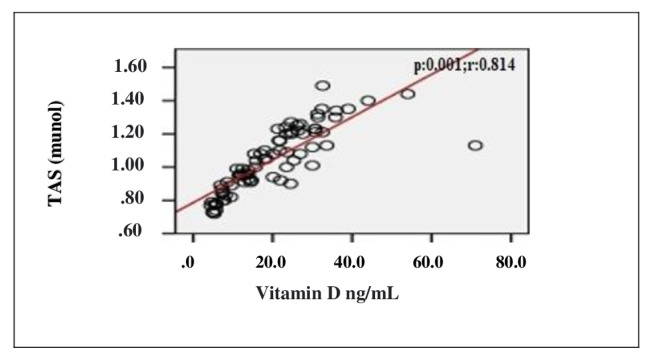
Correlation assessment between vitamin D level and TAS level (n = 77, total subjects). r: Correlation coefficient; TAS: Total antioxidant status.

**Figure 5:**
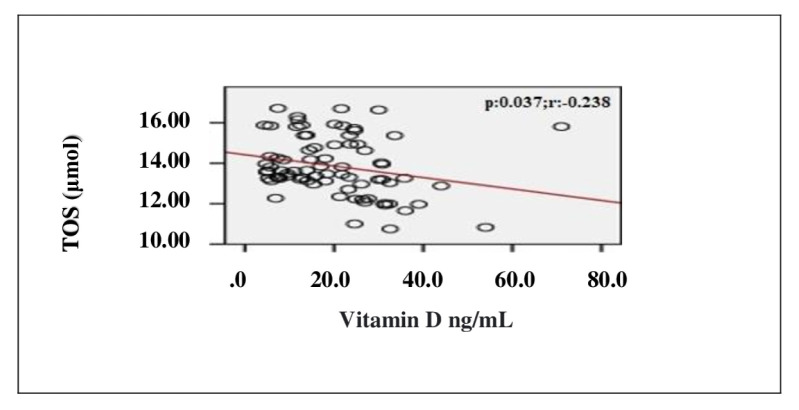
Correlation assessment between vitamin D level and TOS level (n = 77, total subjects). r: Correlation coefficient; TOS: Total oxidant status.

**Figure 6:**
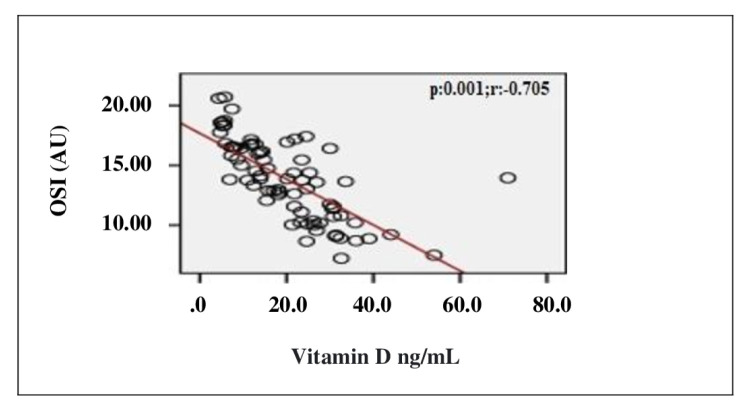
Correlation assessment between vitamin D level and OSI level (n = 77, total subjects). r: Correlation coefficient; OSI: Oxidative stress index.

In the correlation assessment between vitamin D, and age, BMI, adropin, IL-1β, IL-6, TOS, TAS, OSI and other biochemical parameters, significant positive correlation was detected between vitamin D, and adropin and TAS (r = 0.807; p < 0.001, r = 0.814; p < 0.001, respectively). Significant negative correlation was detected between vitamin D, and IL-1β, IL-6, TOS, OSI and PTH (r = −0.725; p < 0.001, r = −0.720; p < 0.001, r = −0.238; p = 0.037, r = −0.705; p < 0.001, r = −0.524; p < 0.001, respectively) (Table 2). Evaluation of the correlation between vitamin D and adropin, IL-1β, IL-6, TOS, TAS and OSI is shown in Figures 1–6.

In the correlation assessment between adropin, and vitamin D, IL-1β, IL-6, TOS, TAS, OSI and PTH, statistically significant positive correlation was detected between adropin and TAS (r = 0.753; p < 0.001), and statistically significant negative correlation was detected between adropin, and IL-1β, IL-6, TOS, OSI and PTH (r = −0.586; p < 0.001, r = −0.659; p < 0.001, r = −0.292; p < 0.010, r = −0.673; p < 0.001, r = −0.511; p < 0.001, respectively) (Table 3). Additionally, comparison of post-hoc analyzes and paired groups is shown in Table 4.

## 4.Discussion

While vitamin D is mainly synthesized in the skin under the influence of sunlight in humans, a small portion is obtained from foods. Vitamin D mainly regulates calcium, phosphorus and bone metabolism, and is also known as an immunomodulator hormone [1–3]. In the literature search, we did not find any study evaluating the relationship between vitamin D level and adropin level.

Our study detected significant difference between the vitamin D groups (G1, G2, G3) in terms of adropin level, and also, a significant positive correlation between vitamin D level and adropin level (p < 0.001). Adropin has been reported to have lipid and glucose homeostasis-regulating, angiogenesis and antiinflammatory effects in addition to preventive effects against insulin resistance and endothelial dysfunction [6,9,10,28,29]. Lovren et al. stated that adropin might have a role in controlling the functions of endothelial cells and protecting the endothelial cells against TNFα-induced apoptosis. They explained this effect of adropin by NOS increase due to increased expression of eNOS via vascular endothelial growth factor receptor (VEGFR2)-phosphatidylinositol 3-kinase-Akt (PI3K-Akt) or VEGFR2-extracellular signal-regulated kinase 1/2 (ERK 1/2) intracellular signal transmission pathways [7,10]. In their study comparing serum adropin levels of a total of 116 type 2 diabetes mellitus (T2DM) patients and 60 control subjects with normal glucose tolerance, Zang H. et al. detected that serum adropin level is lower in T2DM patients, and especially in overweight/obese individuals (compared to the group with normal weight). In the study, it was stated that adropin which is related with glucolipid homeostasis and insulin sensitivity might have a role in the pathogenesis of T2DM [30]. Topuz M et al. compared the serum adropin levels of the groups of subjects with or without endothelial dysfunction using brachial flow-mediated dilation in patients with T2DM. Serum adropin level was detected to be significantly lower in subjects with endothelial dysfunction compared to controls, and it was stated that low adropin level might be a marker of endothelial dysfunction [29]. Plasma adropin levels of individuals with primary hypertension and normotensive individuals were studied in another study. Plasma adropin levels were detected to be significantly lower in the hypertensive group compared to controls, and it was stated that low adropin level might be associated with hypertension [31]. In their study of two groups consisting of individuals with and without CAD, Zhang C et al. found that serum adropin levels are significantly lower in the CAD group compared to controls [32]. Similar to the studies in the literature, in our study, we believe that low serum adropin levels observed in subjects with vitamin D deficiency might trigger the development and progression of endothelial dysfunction, insulin resistance, hypertension and cardiovascular diseases. We believe that the low adropin level in vitamin D deficiency might be caused by the stimulation level of vitamin D receptors in tissues synthesizing adropin and vitamin D affecting the expression of ENHO gene via direct or indirect mechanisms. More studies are needed to illuminate these possible mechanisms.

In our study, significant difference was detected between vitamin D groups in terms of IL-1β and IL-6 levels, and also a significant negative correlation between vitamin D level, and IL-1β and IL-6 levels (p < 0.001). Proinflammatory cytokines (IL-1β, IL-6) have multiple effects on many events including the initiation and maintenance of inflammation, endothelial dysfunction, metabolic syndrome, insulin resistance, diabetes mellitus, oxidative stress and cardiovascular events [18,24–26]. It has been stated that vitamin D might play an important role in the modulation of immune/inflammation system by regulating the production of inflammatory cytokines and inhibiting the proliferation of the proinflammatory cells [33]. In their study comparing coronary heart disease (CHD) patients with their control subjects, Liu Y et al. found that vitamin D level is significantly low in CHD, and detected a significant negative correlation between vitamin D, and IL-1β and IL-6. It has been stated that vitamin D deficiency might induce and aggravate CHD by increasing inflammation via NF-κB [34]. In their study on 60 healthy controls and 106 (59 males, 47 females) T2DM patients, Wang W et al. divided T2DM patients into three groups by vitamin D levels (Vitamin D ≤ 20 ng/mL, 20–30 ng/mL and ≥30 ng/mL). They detected that vitamin D level is considerably lower in T2DM patients compared to healthy controls, and has a negative correlation with IL-1β and IL-6 [35]. On the other hand, in their study in healthy women, Azizieh F. et al. divided the participants into 2 groups by vitamin D levels [as >25 nmol/L (10 ng/mL) and <25 nmol/L]. They did not detect a direct significant relationship between serum vitamin D level, and inflammatory markers, IL-1β and IL-6 [36]. Moreover, Peterson C. A. et al. did not detect a significant relationship and correlation between vitamin D concentrations and IL-6 in women exposed to ultraviolet light (UVB) and divided into two groups as high and low vitamin D level [37]. In their study in type 2 diabetic patients, El Hajj C. et al. did not detect a significant decrease in IL-6 levels following vitamin D replacement treatment compared to prereplacement levels [38]. The results of studies by Liu Y. and Wang W et al. were similar to our results. On the other hand, the results of studies by Azizieh F, Peterson C.A. and El Hajj C. et al. were different from our results. We think that this might be because of the fact that the patient groups in the studies were different in terms of vitamin D level. Accordingly, it suggests that as vitamin D deficiency deepens, the secretion of antiinflammatory cytokines will decrease or the secretion of proinflammatory cytokines will increase leading to worsening of inflammation. Furthermore, it shows that vitamin D can exert its antiinflammatory effects through immune cells by lowering IL-1β and IL-6 levels.

There was a significant negative correlation between vitamin D, and adropin level and oxidative stress marker, TOS, and a significant positive correlation with TAS (p < 0.05). Antioxidants prevent or remove excessive endogenous or exogenous free radical production leading to prevention of free radical-induced cellular damage. The increased oxidative stress as a result of the shift in this balance towards the oxidative stress was detected to play a role in the pathogenesis of various conditions including metabolic syndrome, endothelial dysfunction, diabetes mellitus, hypertension, cardiovascular diseases, malignancy, and kidney and neurological diseases [14,15,18–20]. In their study in patients with vitamin D deficiency and healthy controls, Baser H. et al. detected significant increase in TAS level and significant decrease in TOS level following vitamin D replacement. Moreover, a significant positive correlation was found between vitamin D level TAS, and no significant correlation with TOS [39]. In another study, calcium plus vitamin D supplement for 8 weeks to overweight women with vitamin D deficiency and PCOS has been detected to have beneficial effects on inflammatory factor and biological markers of oxidative stress [40]. In their in vitro study comparing nonenzymatic antioxidants (Vitamin E, melatonin and beta-estradiol) with vitamin D, Lin AM. et al. showed that vitamin D has considerably high antioxidant effect [41]. In a study on the effect of vitamin D on decreasing oxidative stress in diabetes mellitus, vitamin D combined with calcium has been shown to be beneficial in reducing oxidative stress in rats with streptozotocin-induced diabetes [42]. In their study comparing the plasma levels of several enzymatic or nonenzymatic antioxidants in 55 diabetic patients and 40 healthy control subjects, Ramakrishna V. et al. demonstrated that plasma antioxidant levels are considerably lower in patients with DM [43]. Adropin has also been demonstrated to have possible effects on oxidative stress. In their study on the brains of young and older rats, Yang C. et al. suggested that adropin level has negative correlation with endothelial dysfunction and oxidative damage markers, thereby, adropin loss in the brain might play a role in the pathogenesis and development of age-related cerebrovascular dysfunction [44]. Similar to the other studies, our study shows that the low level of vitamin D and adropin changes the oxidative/antioxidative balance in favor of oxidative status (high TOS, low TAS). Increased oxidative stress in vitamin D deficiency might be because of reduced synthesis of NO due to both reduced antioxidant effect of vitamin D and low adropin level. Furthermore, we think that increased oxidative stress might have a trigger role in vitamin D deficiency being a risk factor for many chronic illnesses and metabolic disorders.

This study has several limitations. First, the number of patients included is relatively low and it is a single-center study, and second, it is a female-only study. We think that the strength of our study is the combined evaluation of several parameters related with each other.

## 5. Conclusions

Significantly decreased adropin levels and significantly increased levels of proinflammatory cytokines (IL-1β, IL-6) were detected in vitamin D deficiency with the oxidative/antioxidative balance being changed in favor of oxidative status. Vitamin D could show its effects through vitamin D receptors on tissues via direct or indirect mechanisms. In addition, it may affect adropin release with positive or negative effects on the ENHO gene in adropin secreting tissues. Proinflammatory cytokines, oxidative stress, and adropin targeted studies could contribute to the prevention and treatment of diseases associated with vitamin D deficiency in future. Larger studies are needed to confirm these results.

## Figures and Tables

**Table 1:** Comparison of age, BMI, adropin, IL-1β, IL-6, TOS, TAS, OSI and biochemical parameters of the vitamin D groups.

	Group 1 Vitamin D <20 ng/mL n = 39 Mean ± SD	Group 2 Vitamin D 20–30 ng/mL n = 21 Mean ± SD	Group 3 Vitamin D >30 ng/mL n = 17 Mean ± SD	p
Age (years)	32.35 ± 10.67	35.14 ± 10.85	35.41 ± 7.84	**0.459**
BMI (kg/m^2^)	24.41 ± 5.26	26.70 ± 5.79	23.79 ± 3.81	**0.163**
Adropin (ng/L)	64.34 ± 19.08	109.92 ± 37.81	159.35± 38.65	**<0.001** *
IL-1β (pg/L)	469.65 ± 67.36	326.67 ± 64.32	268.20 ± 35.25	**<0.001** *
IL-6 (ng/L)	143.98 ± 19.46	104.23 ± 12.74	77.41 ± 15.57	**<0.001** *
TOS (μmol)	14.14 ± 1.11	13.95 ± 1.63	13.08 ± 1.66	**0.037** *
TAS (mmol)	0.89 ± 0.10	1.13 ± 0.11	1.26 ± 0.12	**<0.001** *
OSI (AU)	15.95 ± 2.25	12.59 ± 2.69	10.53 ± 2.42	**<0.001** *
Glucose (mg/dL)	91.41 ± 6.52	91.52 ± 6.75	88.47 ± 5.37	**0.240**
Creatinine (mg/dL)	0.70 ± 0.68	0.71 ± 0.52	0.72 ± 0.50	**0.770**
AST (U/L)	17.59 ± 6.51	18.57 ± 5.18	17.11 ± 4.15	**0.716**
ALT (U/L)	16.12 ± 9.48	17.95 ± 9.54	15.47 ± 8.47	**0.677**
ALP (U/L)	62.25 ± 18.40	56.95 ± 17.99	61.94 ± 23.94	**0.586**
GGT (U/L)	16.59 ± 11.38	15.38 ± 7.59	14 ± 4.71	**0.626**
TCholesterol (mg/dL)	179.87 ± 43.13	185.38 ± 34.32	195.94 ± 46.03	**0.417**
Triglyceride (mg/dL)	80.69 ± 43.70	74 ± 28.13	70.52 ± 23.55	**0.585**
LDL-C (mg/dL)	105.10 ± 33.83	104.37 ± 29.34	119.75 ± 36.22	**0.269**
HDL-C(mg/dL)	58.33 ± 11.58	64.37 ± 12.95	59.71 ± 17.37	**0.252**
Ca (mg/dL)	9.42 ± 0.39	9.34 ± 0.40	9.42 ± 0.33	**0.743**
P (mg/dL)	3.64 ± 0.57	3.59 ± 0.35	3.59 ± 0.39	**0.886**
Mg (mg/dL)	1.95 ± 0.14	1.92 ± 0.11	1.94 ± 0.13	**0.757**
PTH (ng/L)	68 ± 19.57	55.16 ± 20.19	38.16 ± 7.11	**<0.001** *
TSH (mU/L)	1.78 ± 0.94	1.53 ± 0.52	1.43 ± 0.51	**0.216**
FT4 (pg/mL)	12.38 ± 1.19	12.37 ± 1.40	12.37 ± 0.83	**0.999**

Mean ± SD: Mean ± standard deviation; BMI: Body mass index; IL-1β: Interleukin-1 beta; IL-6: Interleukin-6; TOS: Total oxidant status; TAS: Total antioxidant status; OSI: Oxidative stress index; AU: Arbitrary unit; AST: Aspartate aminotransferase; ALT: Alanine aminotransferase; ALP: Alkaline phosphatase; GGT: Gamma glutamyl transferase; TCholesterol: Total Cholesterol; LDL-C: Low-density lipoprotein Cholesterol; HDL-C: High density lipoprotein Cholesterol; Ca: Calcium; P: Phosphorus; Mg: Magnesium; PTH: Parathormone; TSH: Thyroid stimulating hormone; FT4: Free tetraiodothyronine;

* Statistical significance (p <0.05).

**Table 2:** Correlation assessment between vitamin D, and age, BMI, adropin, IL-1β, IL-6, TOS, TAS, OSI and biochemical parameters (n = 77, total subjects).

	r	p
Age (years)	−0.006	0.958
BMI (kg/m^2^)	−0.064	0.581
Adropin (ng/L)	0.807	<0.001*
IL-1β (pg/L)	−0.725	<0.001*
IL-6 (ng/L)	−0.720	<0.001*
TOS (μmol)	−0.238	0.037*
TAS (mmol)	0.814	<0.001*
OSI (AU)	−0.705	<0.001*
PTH (ng/L)	−0.524	<0.001*
Calcium (mg/dL)	0.015	0.894

r: Correlation coefficient; BMI: Body mass index; IL-1β: Interleukin-1 beta; IL-6: Interleukin-6; TOS: Total oxidant status; TAS: Total antioxidant status; OSI: Oxidative stress index; AU: Arbitrary unit; PTH: Parathormone;

* Statistical significance (p <0.05).

**Table 3:** Correlation assessment between adropin level, and Vitamin D, IL-1β, IL-6, TOS, TAS, OSI and biochemical parameters (n = 77, total subjects).

	R	p
Vitamin D	0.807	<0.001*
IL-1β (pg/L)	−0.586	<0.001*
IL-6 (ng/L)	−0.659	<0.001*
TOS (μmol)	−0.292	0.010*
TAS (mmol)	0.753	<0.001*
OSI (AU)	−0.673	<0.001*
PTH (ng/L)	−0.511	<0.001*

r: Correlation coefficient; IL-1β: Interleukin-1 beta; IL-6: Interleukin-6; TOS: Total oxidant status; TAS: Total antioxidant status; OSI: Oxidative stress index; AU: Arbitrary unit; PTH: Parathormone;

* Statistical significance (p < 0.05).

**Table 4:** Comparison of post-hoc analyzes and pairwise groups.

	G 1	G2	G3	G1 vs. G2	G1 vs. G3	G2 vs. G3
	Vitamin D < 20 ng/mL n: 39 Mean ± SD	Vitamin D 20–30 ng/mL n: 21 Mean ± SD	Vitamin D >30 ng/mL n: 17 Mean ± SD	P Values	P values	P values
**Adropin (ng/L)**	64.34 ± 19.08	109.92 ± 37.81	159.35 ± 38.65	<0.001	<0.001	<0.001
**IL-1 β (pg/L)**	469.65 ± 67.36	326.67 ± 64.32	268.20 ± 35.25	<0.001	<0.001	0.012
**IL-6 (ng/L)**	143.98 ± 19.46	104.23 ± 12.74	77.41 ± 15.57	<0.001	<0.001	<0.001
**TOS (μmol )**	14.14 ± 1.11	13.95 ± 1.63	13.08 ± 1.66	0.868	0.030	0.148
**TAS (mmol )**	0.89 ± 0.10	1.13 ± 0.11	1.26 ± 0.12	<0.001	<0.001	0.001
**OSI (AU)**	15.95 ± 2.25	12.59 ± 2.69	10.53 ± 2.42	<0.001	<0.001	0.029

G1: Group 1, Vitamin D < 20 ng/mL; G2: Group 2, Vitamin D 20–30 ng/mL; G3: Group 3, Vitamin D > 30 ng/mL; IL-1β: Interleukin-1 beta; IL-6: Interleukin-6; TOS: Total oxidant status; TAS: Total antioxidant status; OSI: Oxidative stress index; AU: Arbitrary unit;

* Statistical significance (p < 0.05).

## References

[1] Kim DH Meza CA Clarke H Kim JS Hickner RC Vitamin D and Endothelial Function Nutrients 2020 12 2 575 10.3390/nu12020575 32098418 PMC7071424

[2] Charoenngam N Holick MF Immunologic Effects of Vitamin D on Human Health and Disease Nutrients 2020 12 7 2097 10.3390/nu12072097 32679784 PMC7400911

[3] Prietl B Treiber G Pieber TR Amrein K Vitamin D and immune function Nutrients 2013 5 7 2502 2521 10.3390/nu5072502 23857223 PMC3738984

[4] Holick MF The vitamin D deficiency pandemic: Approaches for diagnosis, treatment and prevention Reviews in Endocrine and Metabolic Disorders 2017 18 2 153 165 10.1007/s11154-017-9424-1 28516265

[5] Holick MF Vitamin D: a D-Lightful health perspective *Nutrition Reviews* 2008 66 10 Suppl 2 S182 194 10.1111/j.1753-4887.2008.00104.x 18844847

[6] Kumar KG Trevaskis JL Lam DD Sutton GM Koza RA Identification of adropin as a secreted factor linking dietary macronutrient intake with energy homeostasis and lipid metabolism Cell Metabolism 2008 8 6 468 481 10.1016/j.cmet.2008.10.011 19041763 PMC2746325

[7] Zhang S Chen Q Lin X Chen M Liu Q A Review of Adropin as the Medium of Dialogue between Energy Regulation and Immune Regulation Oxidative Medicine and Cellular Longevity 2020 2020 3947806 10.1155/2020/3947806 32190172 PMC7073478

[8] Li L Xie W Zheng XL Yin WD Tang CK A novel peptide adropin in cardiovascular diseases Clinica Chimica Acta 2016 453 107 113 10.1016/j.cca.2015.12.010 26683354

[9] Jasaszwili M Billert M Strowski MZ Nowak KW Skrzypski M Adropin as A Fat-Burning Hormone with Multiple Functions-Review of a Decade of Research Molecules 2020 25 3 549 10.3390/molecules25204757 32012786 PMC7036858

[10] Lovren F Pan Y Quan A Singh KK Shukla PC Adropin is a novel regulator of endothelial function Circulation 2010 14 122 11Suppl S185 192 10.1161/circulationaha.109.931782 20837912

[11] Yosaee S Soltani S Sekhavati E Jazayeri S Adropin-A Novel Biomarker of Heart Disease: A Systematic Review Article Iranian Journal of Public Health 2016 45 12 1568 1576 28053922 PMC5207097

[12] Nergiz S Altinkaya SO Kurt Ömürlü İ Yuksel H Küçük M Circulating adropin levels in patients with endometrium cancer Gynecological Endocrinology 2015 31 9 730 735 10.3109/09513590.2015.1065480 26172926

[13] Oruc CU Akpinar YE Dervisoglu E Amikishiyev S Salmaslıoglu A Low concentrations of adropin are associated with endothelial dysfunction as assessed by flow-mediated dilatation in patients with metabolic syndrome Clinical Chemistry Laboratory Medicine 2017 55 1 139 144 10.1515/cclm-2016-0329 27474839

[14] Burton GJ Jauniaux E Oxidative stress Best Practice Research Clinical Obstetrics Gynaecology 2011 25 3 287 299 10.1016/j.bpobgyn.2010.10.016 21130690 PMC3101336

[15] Young IS Woodside JV Antioxidants in health and disease Journal of Clinical Pathology 2001 54 3 176 186 10.1136/jcp.54.3.176 11253127 PMC1731363

[16] Erel O A new automated colorimetric method for measuring total oxidant status Clinical Biochemistry 2005 38 12 1103 1111 10.1016/j.clinbiochem.2005.08.008 16214125

[17] Erel O A novel automated direct measurement method for total antioxidant capacity using a new generation, more stable ABTS radical cation Clinical Biochemistry 2004 37 4 277 285 10.1016/j.clinbiochem.2003.11.015 15003729

[18] Srikanthan K Feyh A Visweshwar H Shapiro JI Sodhi K Systematic Review of Metabolic Syndrome Biomarkers: A Panel for Early Detection, Management, and Risk Stratification in the West Virginian Population International Journal of Medical Sciences 2016 13 1 25 38 10.7150/ijms.13800 26816492 PMC4716817

[19] Pisoschi AM Pop A The role of antioxidants in the chemistry of oxidative stress: A review European Journal of Medicinal Chemistry 2015 97 55 74 10.1016/j.ejmech.2015.04.040 25942353

[20] Chaudhary P Pandey A Azad CS Tia N Singh M Association of oxidative stress and endothelial dysfunction in hypertension Analytical Biochemistry 2020 590 113535 10.1016/j.ab.2019.113535 31821803

[21] Furukawa S Fujita T Shimabukuro M Iwaki M Yamada Y Increased oxidative stress in obesity and its impact on metabolic syndrome The Journal of Clinical Investigation 2004 114 12 1752 1761 10.1172/JCI21625 15599400 PMC535065

[22] Murakami M Kamimura D Hirano T Pleiotropy and Specificity: Insights from the Interleukin 6 Family of Cytokines Immunity 2019 50 4 812 831 10.1016/j.immuni.2019.03.027 30995501

[23] Garlanda C Dinarello CA Mantovani A The interleukin-1 family: back to the future Immunity 2013 39 6 1003 1118 10.1016/j.immuni.2013.11.010 24332029 PMC3933951

[24] Alexandraki K Piperi C Kalofoutis C Singh J Alaveras A Inflammatory process in type 2 diabetes: The role of cytokines Annals of the New York Academy of Sciences 2006 1084 89 117 10.1196/annals.1372.039 17151295

[25] Aroor AR McKarns S Demarco VG Jia G Sowers JR Maladaptive immune and inflammatory pathways lead to cardiovascular insulin resistance Metabolism 2013 62 11 1543 1552 10.1016/j.metabol.2013.07.001 23932846 PMC3809332

[26] Stienstra R Tack CJ Kanneganti TD Joosten LA Netea MG The inflammasome puts obesity in the danger zone Cell Metabolism 2012 15 1 10 18 10.1016/j.cmet.2011.10.011 22225872

[27] Guler EM Gokce M Bacaksiz A Kocyigit A Urotensin-II, oxidative stress, and inflammation increase in hypertensive and resistant hypertensive patients Clinical and Experimental Hypertension 2021 43 3 211 216 10.1080/10641963.2020.1847128 33172302

[28] Aydin S Kuloglu T Aydin S Eren MN Yilmaz M Expression of adropin in rat brain, cerebellum, kidneys, heart, liver, and pancreas in streptozotocin-induced diabetes Molecular and Cellular Biochemistry 2013 380 1–2 73 81 10.1007/s11010-013-1660-4 23620340

[29] Topuz M Celik A Aslantas T Demir AK Aydin S Plasma adropin levels predict endothelial dysfunction like flow-mediated dilatation in patients with type 2 diabetes mellitus Journal of Investigative Medicine 2013 61 8 1161 1164 10.2310/JIM.0000000000000003 24113736

[30] Zang H Jiang F Cheng X Xu H Hu X Serum adropin levels are decreased in Chinese type 2 diabetic patients and negatively correlated with body mass index Endocrine Journal 2018 65 7 685 691 10.1507/endocrj.EJ18-0060 29669965

[31] Gu X Li H Zhu X Gu H Chen J Inverse Correlation Between Plasma Adropin and ET-1 Levels in Essential Hypertension: A Cross-Sectional Study Medicine (Baltimore) 2015 94 40 e1712 10.1097/MD.0000000000001712 26448026 PMC4616732

[32] Zhang C Zhao L Xu W Li J Wang B Correlation of serum adropin level with coronary artery disease Zhonghua Yi Xue Za Zhi 2014 94 16 1255 1257 24924892

[33] Yin K Agrawal DK Vitamin D and inflammatory diseases Journal of Inflammation Research 2014 7 69 87 10.2147/JIR.S63898 24971027 PMC4070857

[34] Liu Y Peng W Li Y Wang B Yu J Vitamin D Deficiency Harms Patients with Coronary Heart Disease by Enhancing Inflammation Medical Science Monitor 2018 24 9376 9384 10.12659/MSM.911615 30581189 PMC6320654

[35] Wang W Zhang J Wang H Wang X Liu S Vitamin D deficiency enhances insulin resistance by promoting inflammation in type 2 diabetes International Journal of Clinical and Experimental Pathology 2019 12 5 1859 1867 31934009 PMC6947100

[36] Azizieh F Alyahya KO Raghupathy R Association between levels of vitamin D and inflammatory markers in healthy women Journal of Inflammation Research 2016 9 51 57 10.2147/JIR.S103298 27175089 PMC4854309

[37] Peterson CA Heffernan ME Serum tumor necrosis factor-alpha concentrations are negatively correlated with serum 25 (OH) D concentrations in healthy women Journal of Inflammation (London, England) 2008 5 10 10.1186/1476-9255-5-10 18652680 PMC2503979

[38] El Hajj C Walrand S Helou M Yammine K Effect of Vitamin D Supplementation on Inflammatory Markers in Non-Obese Lebanese Patients with Type 2 Diabetes: A Randomized Controlled Trial Nutrients 2020 12 7 2033 10.3390/nu12072033 32659891 PMC7400886

[39] Baser H Can U Baser S Hidayetoglu BT Aslan U Serum total oxidant/anti-oxidant status, ischemia-modified albumin and oxidized-low density lipoprotein levels in patients with vitamin D deficiency Archives of Endocrinology and Metabolism 2015 59 4 318 324 10.1590/2359-3997000000055 26331319

[40] Foroozanfard F Jamilian M Bahmani F Talaee R Talaee N Calcium plus vitamin D supplementation influences biomarkers of inflammation and oxidative stress in overweight and vitamin D-deficient women with polycystic ovary syndrome: a randomized double-blind placebo-controlled clinical trial Clinical Endocrinology 2015 83 6 888 894 10.1111/cen.12840 26119844

[41] Lin AM Chen KB Chao PL Antioxidative effect of vitamin D3 on zinc-induced oxidative stress in CNS Annals of the New York Academy of Sciences 2005 1053 319 329 10.1196/annals.1344.028 16179538

[42] Alatawi FS Faridi UA Alatawi MS Effect of treatment with vitamin D plus calcium on oxidative stress in streptozotocin-induced diabetic rats Saudi Pharmaceutical Journal 2018 26 8 1208 1213 10.1016/j.jsps.2018.07.012 30532641 PMC6260496

[43] Ramakrishna V Jailkhani R Oxidative stress in non-insulin-dependent diabetes mellitus (NIDDM) patients Acta Diabetologica 2008 45 1 41 46 10.1007/s00592-007-0018-3 17924055

[44] Yang C DeMars KM Candelario-Jalil E Age-Dependent Decrease in Adropin is Associated with Reduced Levels of Endothelial Nitric Oxide Synthase and Increased Oxidative Stress in the Rat Brain Aging and Disease 2018 9 2 322 330 10.14336/AD.2017.0523 29896421 PMC5963353

